# Results of Surgical Management of Achilles Tendon Rupture Using the Modified Lindholm Procedure

**DOI:** 10.7759/cureus.9159

**Published:** 2020-07-12

**Authors:** Ali Bilge, Tolgahan Kuru

**Affiliations:** 1 Orthopaedics and Traumatology, Canakkale Onsekiz Mart University, Canakkale, TUR

**Keywords:** achilles tendon, tendon rupture, modified lindholm, surgical procedure

## Abstract

Objectives

Achilles tendon rupture (ATR) in adults often results from sporting activities, especially in young adults. There is no consensus in the literature on the best treatment approach in the treatment of these ruptures. The objective of this study was to evaluate the clinical long-term results of the augmented ATR repair using the modified Lindholm procedure (MLP).

Methods

Patients who underwent MLP due to ATR in the orthopedics and traumatology clinic of our hospital between 2007 and 2014 were retrospectively evaluated. Medical history of the patients was noted, and preoperative physical examination was routinely performed using the Thompson compression test. Patients’ demographic data such as age and gender, tendon rupture side, postoperative follow-up duration, and gap range values were recorded and evaluated.

Results

The mean age of the patients was 29.43 ± 7.10 years. The mean postoperative follow-up duration was 50.1 ± 8.20 months. Of the patients, 16 were injured during football, 10 during basketball, 2 during volleyball, and the remaining 8 during other sporting activities. When Arner-Lindholm scores were evaluated during follow-up, excellent outcome was achieved in 30 patients and good outcome was achieved in 6 patients, whereas there was no patient with poor outcome. None of the patients developed tendon re-rupture. At the end of the fourth postoperative month, the range of ankle motion was 100% in all patients.

Conclusions

In patients with spontaneous AT tendon rupture, MLP seems to prevent the re-rupture in the long-term period and should be considered as a safe procedure to repair ATR.

## Introduction

Achilles tendon (AT) is one of the most commonly ruptured tendons in the body and typically affects patients between 35 and 60 years of age [1]. AT rupture (ATR) in adults often result from spontaneous jumping activities, without specific traumatic event [2,3]. Historically, Quenu and Stoinovitch stated that "rupture of the AT should be operated on without delay" [4]. Arner et al. compared the results of the operative and conservative treatment of ATR and found better results in the former group [5]. Following their work, surgical treatment became the mainstay for treating adults with ATR to avoid the risk of re-rupture, AT incompetence, and gait disturbances [3,6,7]. Möller et al. compared outcomes of non-operative and operative treatment of ATR in 112 patients and reported a higher rate of re-rupture in the non-operative group (11/53) compared with the operative group (1/59) [7]. Non-operative treatment has been preferred for the elderly patients [2]. Operative techniques can be classified into open and percutaneous ones [8-11].

In a comparison of percutaneous and open surgical repairs, the cosmetic results were better with percutaneous procedures, and recovery of strength and motion were essentially the same as for both, but the rate of re-rupture was higher in percutaneous cases [12].

Since ATR is a severe type of injury and one of the most common tendinous lesions, naturally many treatment procedures have been reported [13]. In their quantitative analysis of operative treatments for AT, Wong et al. recognized at least 41 different open techniques [14]. Open operative techniques can be divided mainly into treatments with augmentation and those without augmentation. Primary repair using the simple end-to-end modified Kessler techniques has been utilized by many centers. Lindholm et al. developed an augmentation method that reinforces the repair and prevents adhesion to the overlying skin. Repair augmentation using the Lindholm technique as an alternative to primary end-to-end repair has been used to get stronger repairs [15].

Re-rupture following the primary end-to-end repairs has been reported previously. The modified Lindholm procedure (MLP) as an augmented ATR repair with turn-down of gastrocnemius flap will reduce the re-rupture rates [16]. The objective of this study was to evaluate the clinical long-term results of the augmented ATR repair using MLP.

## Materials and methods

Patients who underwent MLP due to ATR in the orthopedics and traumatology clinic of our hospital between 2007 and 2014 were retrospectively evaluated. Data of the patients were obtained from the hospital registry system. Patients with a history of previous AT surgery, fractures around the ankle and distal tibia, systemic connective tissue disorders, and open injuries in the AT were excluded from the study.

Medical history of the patients was noted, and preoperative physical examination was routinely performed using the Thompson compression test. Typical complaints of the patients included pain, difficulty in walking, and inability to running. Anterior-posterior and lateral radiographs of the patients were examined in order to rule out avulsion fractures. Patients’ demographic data such as age and gender, tendon rupture side, postoperative follow-up duration, and gap range values were recorded. In addition, dorsiflexion and plantar flexion ranges of the operated ankle were evaluated using the Arner-Lindholm scale (Table 1) [5].

**Table 1:** Arner-Lindholm Scale for Achilles Tendon ROM, range of motion; PF, plantar flexion; DF, dorsiflexion

Excellent
Free from discomfort and essentially normal function
Normal walking power, tip toe, calf muscle power
Calf circumference < 1 cm
Ankle ROM degree < 5 degrees (PF/DF)
Good
Mild discomfort
Slightly decreased walking power, tip toe, calf muscle power
Calf circumference < 3 cm
Ankle ROM degree < 15 degrees (PF/DF)
Poor
Dissatisfied or marked discomfort
Limp, inability to tip toe
Calf circumference > 3 cm
Ankle ROM: DF decrease > 10 degrees or PF decrease > 15 degrees (PF/DF)

Clinical follow-up data were obtained by evaluation of the patients by an independent surgeon. The study was conducted in accordance with the principles of the Declaration of Helsinki.

Surgical technique

**Figure 1:**
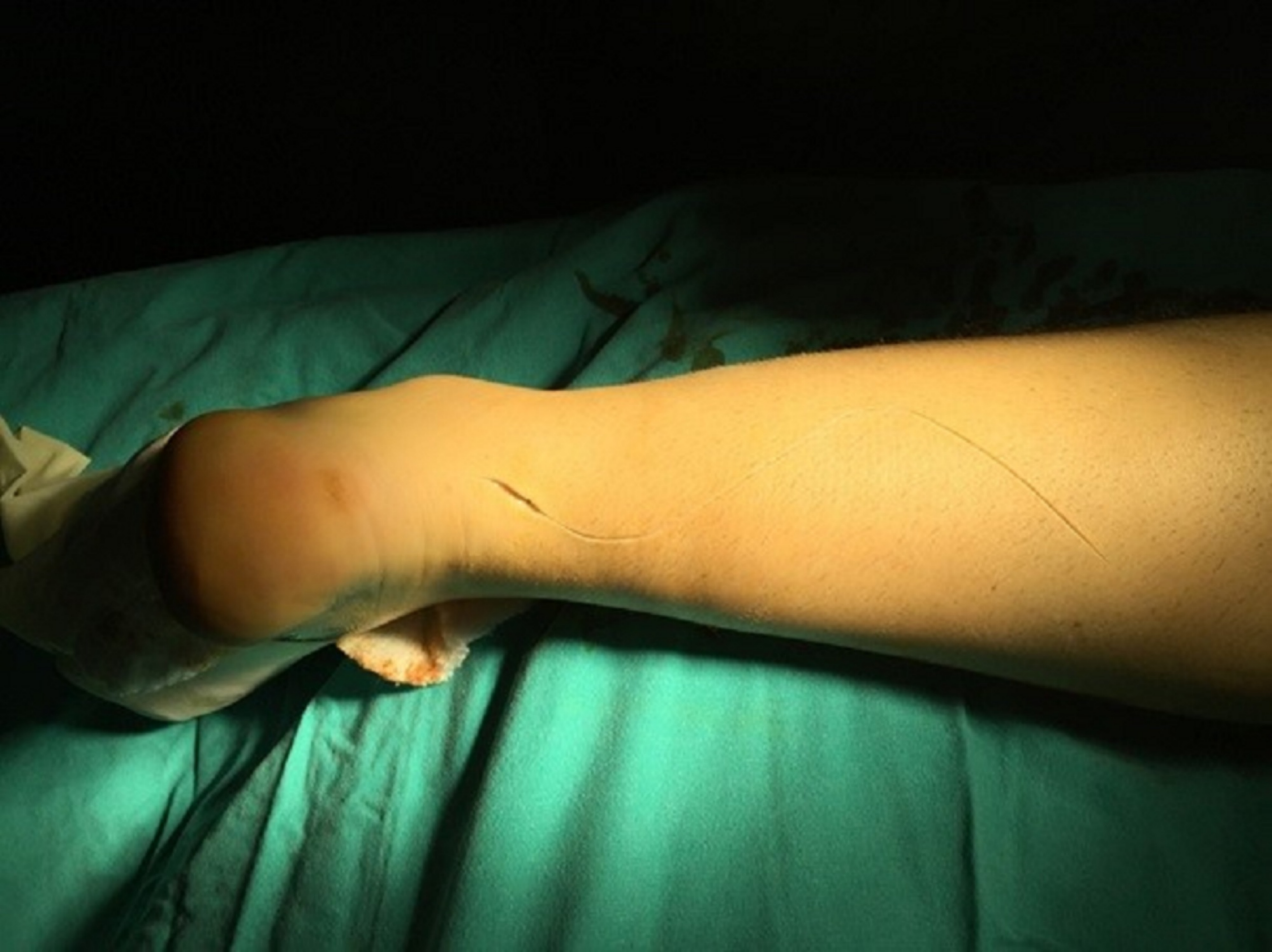
S-shaped incision

The MLP operative technique used in this study has been described previously [17]. In our MLP experience, we used an S-shaped incision to prevent post-operative adhesion and contractures (Figure 1).

**Figure 2:**
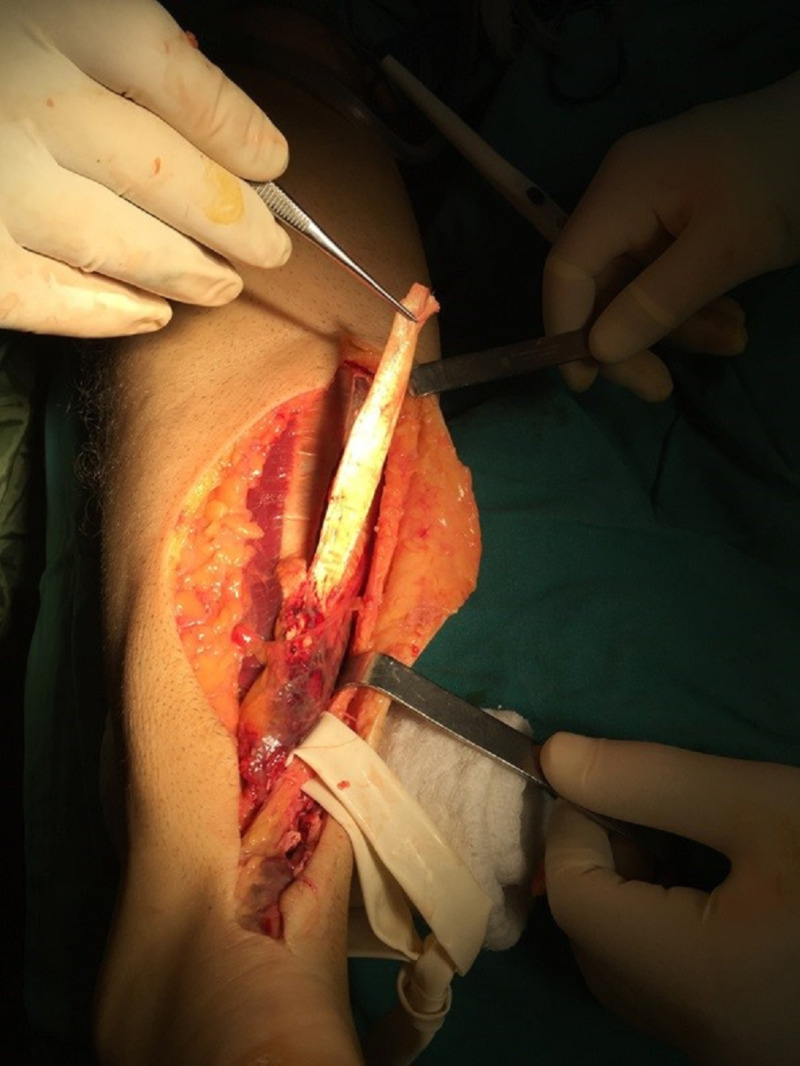
Strip of the gastrocnemius tendon

A single strip of the gastrocnemius tendon was prepared (Figure 2).

**Figure 3:**
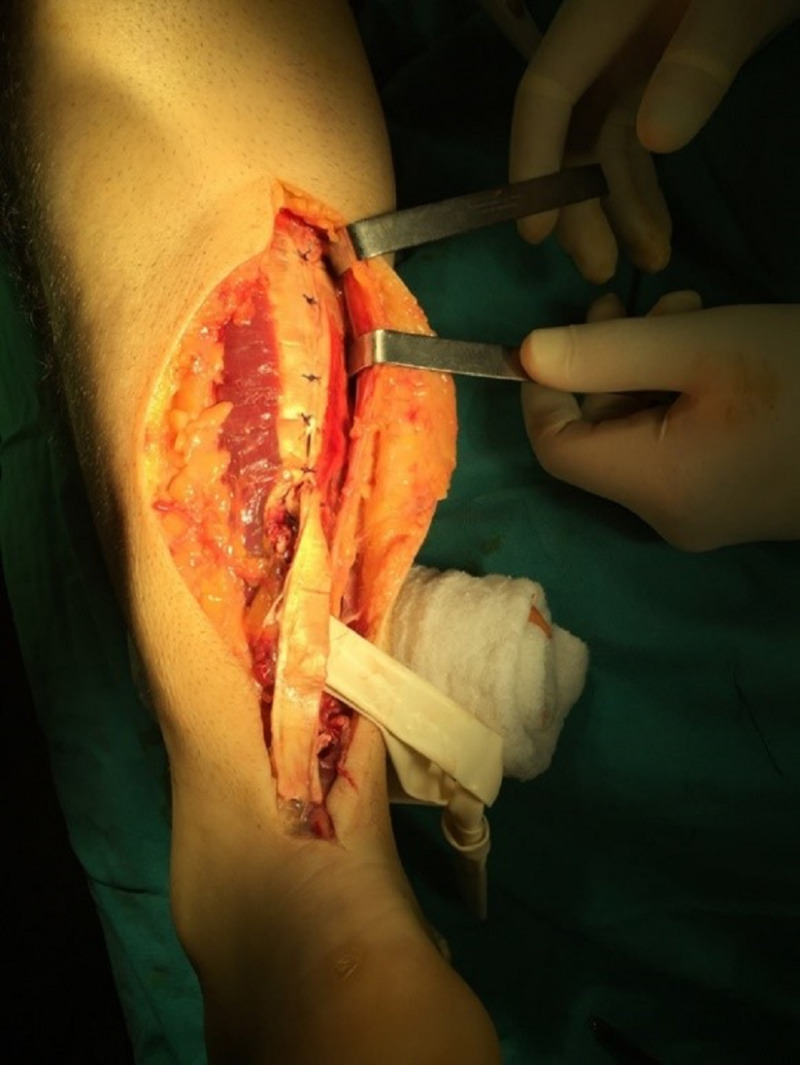
Turn down strip of the gastrocnemius tendon

The two ends of the AT were repaired with a locking modified Kessler technique and then the suture line was reinforced with a turn-down strip of the gastrocnemius tendon (Figure 3).

Hemostasis was performed in all patients, and the skin was sutured using absorbable sutures. A short leg cast was applied in the gravity equinus position for the first four postoperative weeks and then it was replaced by neutral position over the next four weeks. Weight-bearing was not allowed in the eight postoperative weeks.

Statistical analysis

Data obtained from the study were statistically analyzed using SPSS Version 20.0 (IBM Corp., Armonk, USA). Continuous variables are expressed as mean ± standard deviation (minimum and maximum values). Categorical variables are expressed as number and percentage.

## Results

A total of 36 patients underwent MLP due to ATR in our clinic between 2007 and 2014. All of the patients included in the study had ATR during sporting activities such as football, basketball, and volleyball. The mean age of the patients was 29.43 ± 7.10 (min-max: 22-44) years. Of the patients, 34 (94.5%) were males and 2 (5.5%) were females. The maximum duration from the time of injury to intervention was two weeks, but majority (83.3%) of the patients were operated within the 10th day of the injury. The mean postoperative follow-up duration was 50.1 ± 8.20 months (min-max: 24-72 months). The mean length of ATR was measured as 4.2 ± 0.80 cm (min-max: 2-6 cm). Of the 36 patients, 16 (44.4%) were injured during football, 10 (27.7%) during basketball, 2 (5.5%) during volleyball, and the remaining 8 (22.4%) during other sporting activities.

When Arner-Lindholm scores during follow-up were evaluated, excellent outcome was achieved in 30 (83.3%) patients and good outcome was achieved in 6 (16.7%) patients, whereas there was no patient with poor outcome (Table 2).

**Table 2:** Distribution of Arner-Lindholm Scores

Arner-Lindholm Scores	n	%
Excellent	30	83.3
Good	6	16.7
Poor	0	0

One patient required foreign body granuloma resection at the eighth postoperative month. Another patient developed skin necrosis at the edge of the wound and required debridement under local anesthesia. None of the patients developed tendon re-rupture. At the end of the fourth postoperative month, range of ankle motion was 100% in all patients. All patients returned to daily activities, whereas 25 (73.5%) returned to their pre-injury sport activities.

## Discussion

The best treatment method to use in the treatment of ATR is controversial in the literature. When determining the treatment method, the need of individual patient should be taken into account according to the patient’s age, comorbidity, functional requirements, and activities. Surgical treatment has been associated with various complications such as scar adhesions and infections. Some studies have demonstrated that non-surgical treatment approaches show similar outcomes with surgical methods, and these approaches are indicated especially in high-risk patients [18,19]. On the other hand, athletes are in general younger and more healthy with low rate of comorbidities. Various studies have shown that generally surgical repair provides earlier return to sporting activities and lower rate of re-rupture [14,20]. However, the risk of re-rupture increases in non-operative treatment of ATR. Once tendon is ruptured, it cannot bear the intensity of pre-injury activities. All patients in our study were injured during sporting activities, and the mean age of the patients was 29.43 years. Therefore, in our study we used surgical methods in our patients in line with the opinions in the literature. In our study, the number of male patients was significantly higher than that of female patients. This was attributed to that men are more commonly involved in active sports compared with women.

Several methods have been described in the literature for surgical repair of ATR. Clinical results following the primary repair of ATR have been reported in studies. However, primary repair using the Kessler method alone without augmentation subjects patients to the risk of re-rupture [3-5]. These concerns as well as the difficulties in second surgeries have driven surgeons to develop augmentation of the primary repair using the gastrocnemius flaps for ATR.

While repair augmentation techniques have superior biomechanical properties [16], data regarding long-term prevention of re-rupture are lacking in the previous studies. In this study, 36 adult patients underwent MLP. No patient had a re-rupture requiring further surgeries. We used the augmentation technique with single central slip of AT because of the better coverage of the rupture site. Better coverage might minimize adhesion by allowing a more physiological excursion. We observed that this result was likely due to native tissue coverage around the ruptured tendon. In an effort to prevent adhesion into the skin and subcutaneous tissues, we used an S-shaped skin incision. We believe that the S-shaped incision affords the advantage of avoiding direct contact between the skin suture and the rupture site and improves postoperative range of motion [5,12,15].

In our study, the mean follow-up duration was 50.1 months. Similarly, in a study by Demirel et al. using a similar surgical method, the mean follow-up duration was reported as 55.2 months [21]. Also, in a study by Garabito et al. the mean follow up duration was 57.6 months [22]. In this study, only one patient developed skin necrosis at the edge of the incision requiring observation, and none of the patients developed re-rupture.

Study limitations

This study has some limitations. First, the study has a retrospective design. Second, the number of patients was relatively small as ATR is rarely seen and the study was conducted in a single center. Finally, we could not compare our method with the methods used in other studies. However, given that studies on surgical repair of ATR are limited, our results would provide a significant contribution to the existing evidence.

## Conclusions

MLP is an effective surgical option for the management of ATR in adults and minimizes or eliminates the re-rupture concerns of pure primary repairs without augmentation. The S-shape surgical skin incision improves range of motion by preventing adhesions.

In patients with spontaneous ATR, MLP seems to prevent the re-rupture in the long-term period and should be considered as a safe procedure to repair ATR. Further multicenter studies should be conducted in order to support our findings.
